# Neoadjuvant therapy of metformin is associated with good tumor response after preoperative concurrent chemoradiotherapy for rectal cancer

**DOI:** 10.1038/s41598-022-07768-2

**Published:** 2022-03-08

**Authors:** Jeonghee Han, Jong Ho Kim, Jin-Won Lee, Sang Hyup Han, Haesung Kim

**Affiliations:** 1 Department of Surgery, Chuncheon Sacred Heart Hospital, Hallym University College of Medicine, Chuncheon, 24253 Republic of Korea; 2 Department of Pharmacology, Kangwon University College of Medicine, Chuncheon, Republic of Korea; 3 Department of Anesthesiology and Pain Medicine, Chuncheon Sacred Heart Hospital, Hallym University College of Medicine, Chuncheon, 24253 Republic of Korea

**Keywords:** Diseases, Oncology

## Abstract

Metformin is associated with good tumor response in preoperative concurrent chemoradiotherapy (CCRT) for rectal cancer. This study aims to demonstrate that the timing of metformin is related to the tumor response on preoperative CCRT for rectal cancer. From January 2010 to December 2017, 232 patients who underwent curative resection after preoperative CCRT were reviewed. Patients were divided into groups with or without diabetes or metformin. The timing of metformin administration was divided based on before and from initiation of CCRT. Multivariate logistic regression analysis was used to identify predictors for tumor response. Tumor downstaging (*p* = 0.02) and good response rates of tumor regression grade (TRG) (*p* = 0.008) were significantly higher in the group administered metformin before CCRT than other groups. In the multivariate analysis, metformin administration before CCRT was a significant factor in predicting tumor downstaging [odds ratio (OR) 10.31, 95% confidence interval (CI) 1.76–102.08, *p* = 0.02] and good TRG (OR 12.55, 95% CI 2.38–80.24, *p* = 0.004). In patients with rectal cancer who underwent preoperative CCRT, neoadjuvant therapy of metformin before CCRT was significantly associated with good tumor response.

## Introduction

Worldwide, the incidence of colorectal cancer is gradually increasing. The third most common disease is colorectal cancer among various carcinomas, and its cancer-specific mortality rate is the fourth^1^. Preoperative concurrent chemoradiotherapy (CCRT) is crucial for the treatment of locally advanced rectal cancer. Thanks to preoperative CCRT for rectal cancer, it is possible to expect the effect of reducing the stage of tumor or lymph nodes and achieving pathological complete response (pCR) of the tumor^2^. Besides, pCR after CCRT correlates with better postoperative survival in patients with rectal cancer^3^. However, the benefit of pCR can only be observed in less than 20% of patients, and the reason for the difference in tumor response after CCRT is still unknown^4^.

Metformin is a standard treatment for type 2 diabetes mellitus (T2DM), and some studies have reported its effectiveness as an anticancer therapy and anticancer adjuvant for various cancers^5,6^. Several meta-analyses have reported that metformin increases the survival rate of colon cancer^6–8^. Several recent studies show that metformin improves tumor response after preoperative CCRT for rectal cancer^9,10^. The effect of metformin on increasing the survival rate of rectal cancer is controversial, but the association between metformin and good tumor response after preoperative CCRT is increasingly emerging^9,10^.

However, the mechanism of metformin on preoperative CCRT for rectal cancer is not yet definite. There is no study on the timing or dose of metformin for preoperative CCRT. Therefore, this study aims to demonstrate whether there is a difference in the tumor response between the metformin usage and the non-metformin usage, and what timing of metformin is associated with a good tumor response as an intervention after preoperative CCRT for rectal cancer. In addition, this study aims to investigate the association between the timing of metformin and survival.

## Methods

### Patients

**Figure 1:**
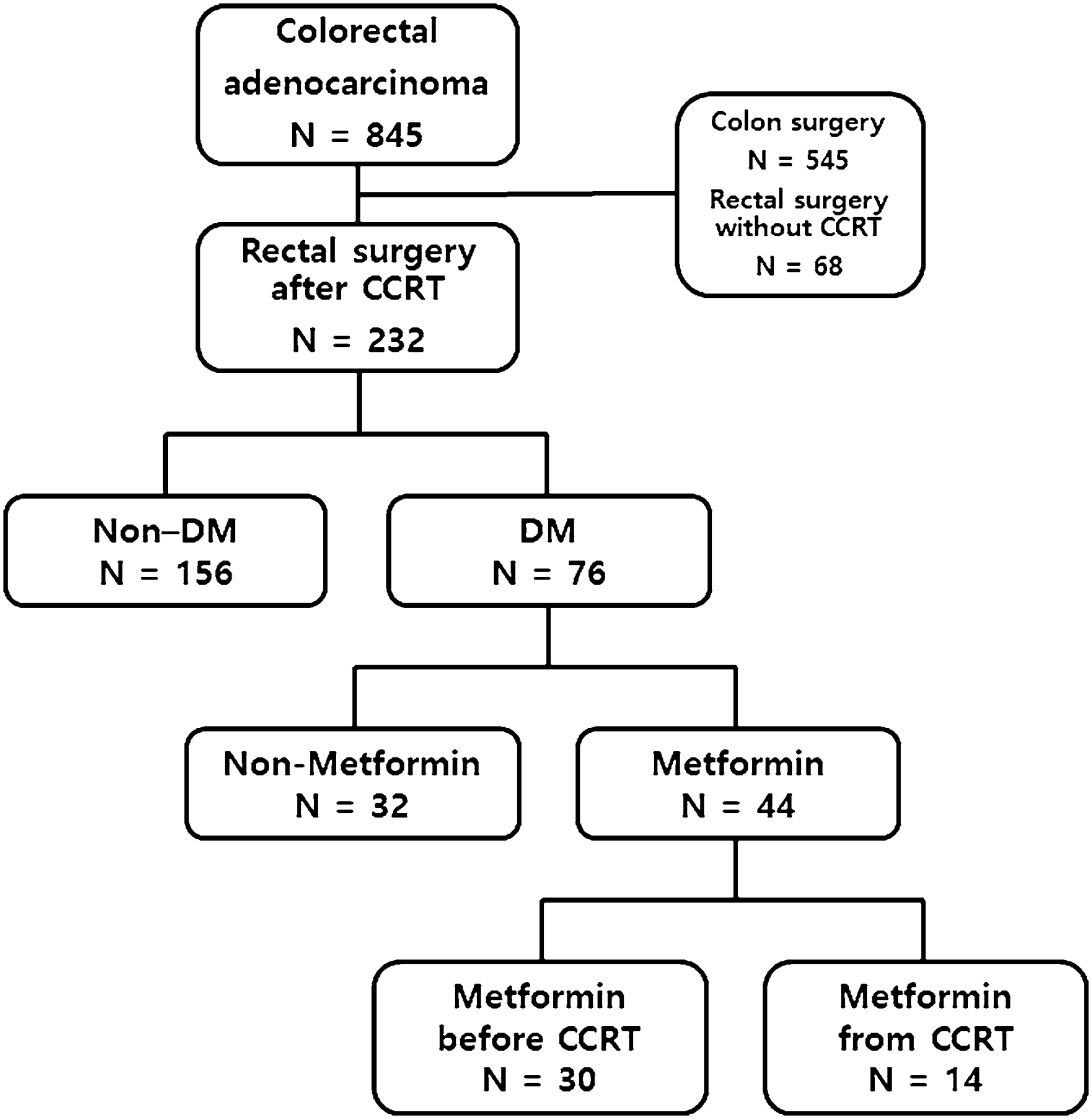
Flow chart of patient group.

Patients diagnosed with rectal adenocarcinoma between January 2010 and December 2017 were retrospectively reviewed. Patients who underwent CCRT were divided into the non-DM group, the group taking non-metformin for DM, the group taking metformin before CCRT for DM, and the group taking metformin from the beginning of CCRT for DM. The timing of metformin administration was divided based on before and from the initiation of CCRT (Fig. 1). DM patients were diagnosed by an endocrinologist. Patients taking metformin from the beginning of CCRT were those with a new diagnosis of DM at the time of diagnosis of rectal cancer. None of the patients stopped taking metformin. Compliance with metformin was checked with continuous follow-up by an endocrinologist. The patient's clinical characteristics included the following factors: age, sex, body mass index (BMI), American Society of Anesthesiologists (ASA) score, metformin dose, preoperative carcinoembryonic antigen (CEA), a hemoglobin A1c (HbA1c), clinical stage, mrT stage, mrN stage, extramural vascular invasion (EMVI), the circumferential resection margin (CRM) status, surgical method, the time interval between CCRT and surgery, tumor histology, and pathology. HbA1c test shows what the average amount of glucose attached to hemoglobin has been over the past three months. If HbA1c levels are high, it may be a sign of diabetes. The rectal tumor was defined as a lesion within 15 cm from the anal verge. The rectum can be divided into three parts: the upper, middle, and lower rectum. From the anal verge, these three parts are defined as follows: the lower rectum, 0–6 cm; the middle rectum, 7–11 cm; and the upper rectum, 12–15 cm. Tumor distance was defined as the distance from the inferior margin of the tumor to the anal verge as measured via colonoscopy. At MRI, CRM status can be obtained by measuring the shortest distance between the outermost part of the rectal tumor and the mesorectal fascia (MRF). The mrCRM status is threatened if it is less than 2 mm. EMVI is an extension of the tumor to the vessels in the mesorectum, resulting in wall irregularity, focal enlargement, and/or signal intensity of the tumor within the vessel at MRI. According to the AJCC 7th stage system, the clinical stage cT 3–4 or cN 0–2 without distant metastasis was the target of preoperative CCRT. The clinical stage was evaluated using endoscopy, ultrasonography, contrast-enhanced helical computed tomography (CT), magnetic resonance imaging (MRI), and positron emission tomography (PET)-CT. Patients with a previous history of cancer, familial genetic cancer syndromes, simultaneous cancer in another organ, distant metastasis, drop out of preoperative CCRT, local excision of the tumor, and no available follow-up data were excluded. This study followed the Declaration of Helsinki and was approved by the Internal Research Board of Chun-Cheon Sacred Heart Hospital (approval number: 2021-05-001). According to the Clinical Ethics Committee of Hallym University College of Medicine, no written consent was required for this retrospective analysis of anonymous data.

### Treatment

Patients had treatment with long course radiation therapy for 5 weeks for preoperative CCRT. The whole irradiation dose was 44–54 Gy, and 1.8–2.0 Gy was irradiated per day. Chemotherapy was administered concurrently with radiation therapy, and the regimen was based on 5-FU or capecitabine. 5-FU (425 mg/m^2^/day) and leucovorin (20 mg/m^2^/day) were infused for 5 days in the first and fifth weeks of radiation therapy. Capecitabine (825 mg/m^2^/day) was administered orally twice daily during radiotherapy. Curative surgery with total mesorectal excision (TME) was performed after 8 weeks after termination CCRT. The time interval between CCRT and surgery does not include consolidation chemotherapy. After surgery, adjuvant chemotherapy was administered. All patients in our study achieved adjuvant chemotherapy to the end.

### Tumor response and survival

Rectal MRI was performed before and after radiotherapy, respectively. The size of the tumor and lymph nodes was measured by rectal MRI. The size of the tumor was measured with the longest diameter. For the size of the lymph node, the short axis diameter of the largest lymph node was chosen^11^. Lymph nodes larger than 10 mm in size were defined as lymph node metastasis in our study. After radical surgery, the pathology was confirmed by pathologists. Tumor responses after CCRT were classified according to Mandard grade. The Mandard grade divided the tumor response into 5 categories according to histomorphological regression: grade 1, fibrosis without detectable tumor tissue (pCR); grade 2, fibrosis with scattered tumor cells; grade 3, fibrosis and tumor cells with a preponderance of fibrosis; grade 4, fibrosis and tumor cells with a preponderance of tumor cells; grade 5, tumor tissue without regression-related changes. Pathological tumor regression grade (pTRG) to CCRT was graded as good (Mandard grade 1, 2) or poor (Mandard grade 3–5)^12^. T-downstaging was defined as the difference between the image before CCRT and the pathology of the final T stage^13^. N-downstaging was defined as the reduction between the pre-CCRT imaging results and the final pathological lymph nodes state^13^. Overall survival (OS) was analyzed between groups. OS was defined as the interval between surgery and death or last follow-up.

### Statistical analysis

The primary endpoint was to determine whether pathological tumor response was correlated with the timing of metformin administration. The secondary endpoint was to determine the difference in overall survival (OS) rates between groups. The data was expressed as mean ± standard deviation for continuous variables and mean ± 95% confidence interval (CI) for categorical variables. The groups were compared regarding clinicopathological factors. Differences between categorical variables were compared using the χ^2^ test. A one-way analysis of variance (ANOVA) or the Kruskal–Wallis rank-sum test was used to assess continuous variables according to the Shapiro–Wilk normality test, followed by post-hoc Tukey’s honestly significant difference multiple comparison test. The Kaplan–Meier method was used to estimate OS, and the log-rank test was used for comparison. The associations between tumor responses and clinicopathologic factors were assessed by using logistic regression analysis. Each factor was assessed in a separate univariate logistic regression analysis. Independent variables reaching a cut-off *p* value of < 0.2 in univariate analyses were considered for inclusion in a multivariable logistic regression model, and an odds ratio (OR) and 95% CI were calculated for each factor. *p* value lower than 0.05 was considered statistically significant. All statistical analyses were performed using SPSS version 25.0 (IBM Corp., Armonk, NY, USA) after removing patient identifiers from all data sets.

## Results

### Patients characteristics

A total of 232 patients were included, with 176 (75%) men and 56 women (24%). There were 156 patients in the non-diabetic group and 76 patients in the diabetic group. In the diabetic group, 32 patients received non-metformin. 30 patients had received metformin before the start of CCRT; administration of metformin was at least 2 weeks before initiation of treatment, and 14 patients received metformin from the initiation of CCRT. There were significant differences in ASA classification between groups (*p* = 0.02). Lower HbA1C (5.3%, *p* < 0.001) was observed in the non-diabetic group. Other clinicopathological features did not show significant differences between groups. Clinicopathological characteristics are summarized in Table 1. The mean dose of metformin was 1159 mg (median: 1000 [250–4000] mg). Non-metformin drugs included insulin, sulfonylureas, thiazolidinediones, an alpha-glucosidase inhibitor, or a dipeptidyl peptidase 4 (DPP-4).

**Table 1:** Clinicopathologic characteristics.

	Non-diabetic N = 156	Non-Metformin N = 32	Metformin Before CCRT N = 30	Metformin from CCRT N = 14	*p* value
**Sex**					0.199
Female	42 (26.9%)	2 (6.2%)	6 (20.0%)	6 (42.9%)	
Male	114 (73.1%)	30 (93.8%)	24 (80.0%)	8 (57.1%)	
Age, y	62.1 ± 11.8	61.8 ± 10.9	64.6 ± 8.7	69.9 ± 5.1	0.101
BMI, kg/m^2^	23.2 [21.2;25.0]	23.2 [20.9;25.9]	23.6 [21.9;25.1]	23.7 [22.8;28.6]	0.689
HbA1c, %	5.3 [ 5.0; 5.8]	6.8 [ 5.8; 7.9]	6.7 [ 6.3; 7.2]	7.3 [ 6.1; 7.5]	< 0.001
Metformin dose, mg			1000.0 [625.0;1500.0]	1000.0 [750.0;1250]	< 0.001
**ASA classification**					0.029
I	26 (16.7%)	0 (0.0%)	0 (0.0%)	0 (0.0%)	
II	106 (67.9%)	22 (68.8%)	16 (53.3%)	10 (71.4%)	
III	23 (14.7%)	10 (31.2%)	12 (40.0%)	4 (28.6%)	
IV	1 (0.6%)	0 (0.0%)	2 (6.7%)	0 (0.0%)	
Pre-CCRT CEA, ng/ml	2.8 [ 1.9; 4.1]	2.3 [ 1.9; 3.8]	3.5 [ 2.8; 8.4]	2.9 [ 2.0; 4.5]	0.435
Post-CCRT CEA, ng/ml	1.8 [ 1.1; 2.7]	1.5 [ 1.2; 2.0]	2.4 [ 1.6; 4.0]	2.6 [ 1.5; 3.1]	0.239
**Tumor location**					0.310
Lower	78 (50.0%)	18 (56.2%)	8 (26.7%)	2 (14.3%)	
Mid	35 (22.4%)	6 (18.8%)	8 (26.7%)	6 (42.9%)	
Upper	43 (27.6%)	8 (25.0%)	14 (46.7%)	6 (42.9%)	
**Operative method**					0.656
Laparoscopic	136 (87.2%)	30 (93.8%)	22 (73.3%)	14 (100.0%)	
Open	3 (1.9%)	0 (0.0%)	0 (0.0%)	0 (0.0%)	
Robot	17 (10.9%)	2 (6.2%)	8 (26.7%)	0 (0.0%)	
**Clinical stage**					0.93
Stage II	65 (41.7%)	16 (50.0%)	12 (40.0%)	6 (42.9%)	
Stage III	91 (58.3%)	16 (50.0%)	18 (60.0%)	8 (57.1%)	
**mrT**^**†**^					0.047
3	153 (98.1%)	30(93.8%)	26 (86.7%)	12 (85.7%)	
4	3 (1.9%)	2 (6.2%)	4 (13.3%)	2 (14.3%)	
**mrN**^**‡**^					0.987
0	65 (41.7%)	16 (50.0%)	12 (40.0%)	6 (42.9%)	
1	22 (14.1%)	2 (6.2%)	4 (13.3%)	2 (14.3%)	
2	69 (44.2%)	14 (43.8%)	14 (46.7%)	6 (42.9%)	
**mrEMVI**					0.781
Negative	125 (80.1%)	28 (87.5%)	26 (86.7%)	10 (71.4%)	
Positive	31 (19.9%)	4 (12.5%)	4 (13.3%)	4 (28.6%)	
**mrCRM threatened**					0.34
Negative	61 (39.1%)	16 (50.0%)	18 (60.0%)	4 (28.6%)	
Positive	95 (60.9%)	16 (50.0%)	12 (40.0%)	10 (71.4%)	
Dose of radiation, cGy	4984.2 ± 194.5	5028.8 ± 254.5	4985.3 ± 229.6	5080.0 ± 183.3	0.296
Histologic grade*					0.578
High	10 (6.4%)	4 (12.5%)	4 (13.3%)	2 (14.3%)	
Low	146 (93.6%)	28 (87.5%)	26 (86.7%)	12 (85.7%)	
**VI**					0.792
Negative	130 (83.3%)	28 (87.5%)	26 (86.7%)	10 (71.4%)	
Positive	26 (16.7%)	4 (12.5%)	4 (13.3%)	4 (28.6%)	
**LI**					0.407
Negative	131 (84.0%)	28 (87.5%)	30 (100.0%)	12 (85.7%)	
Positive	25 (16.0%)	4 (12.5%)	0 (0.0%)	2 (14.3%)	
ypCRM*^†§^					0.433
Negative	154 (98.7%)	30 (93.8%)	30 (100.0%)	14 (100.0%)	
Positive	2 (1.3%)	2 (6.2%)	0 (0.0%)	0 (0.0%)	
**Interval to surgery**					0.09
≤ 8 wk	149 (95.5%)	28 (87.5%)	26 (86.7%)	12 (85.7%)	
> 8 wk	7 (4.5%)	4 (12.5%)	4 (13.3%)	2 (14.3%)	
**Adjuvant chemotherapy**					0.434
FL	76 (48.7%)	12 (37.5%)	20 (66.7%)	6 (42.9%)	
FOLFOX	80 (51.3%)	20 (62.5%)	10 (33.3%)	8 (57.1%)	

Data are n (%) or mean ± SD., Continuous variables expressed as median and interquartile range (IQR 25%–75%).

ASA = American Society of Anesthesiologists, VI = vascular invasion, LI = lymphatic invasion, CEA = carcinoembrionic antigen, BMI = body mass index, HbA1c = hemoglobin A1c, mrEMVI = Magnetic resonance imaging extramural vascular invasion. mrCRM = Magnetic resonance imaging circumferential resection margin, FL = 5-FU + leucovorin, FOLFOX = 5-FU + oxaliplatin. mrT^†^ = Magnetic resonance imaging Tumor stage; mrN^‡^ = Magnetic resonance imaging stage; Histologic grade* Low = well or moderately differentiated; High = poorly differentiated or mucinous carcinoma; ypCRM*^†§^ = Pathological circumferential resection margin.

### Timing of metformin and tumor response

**Figure 2:**
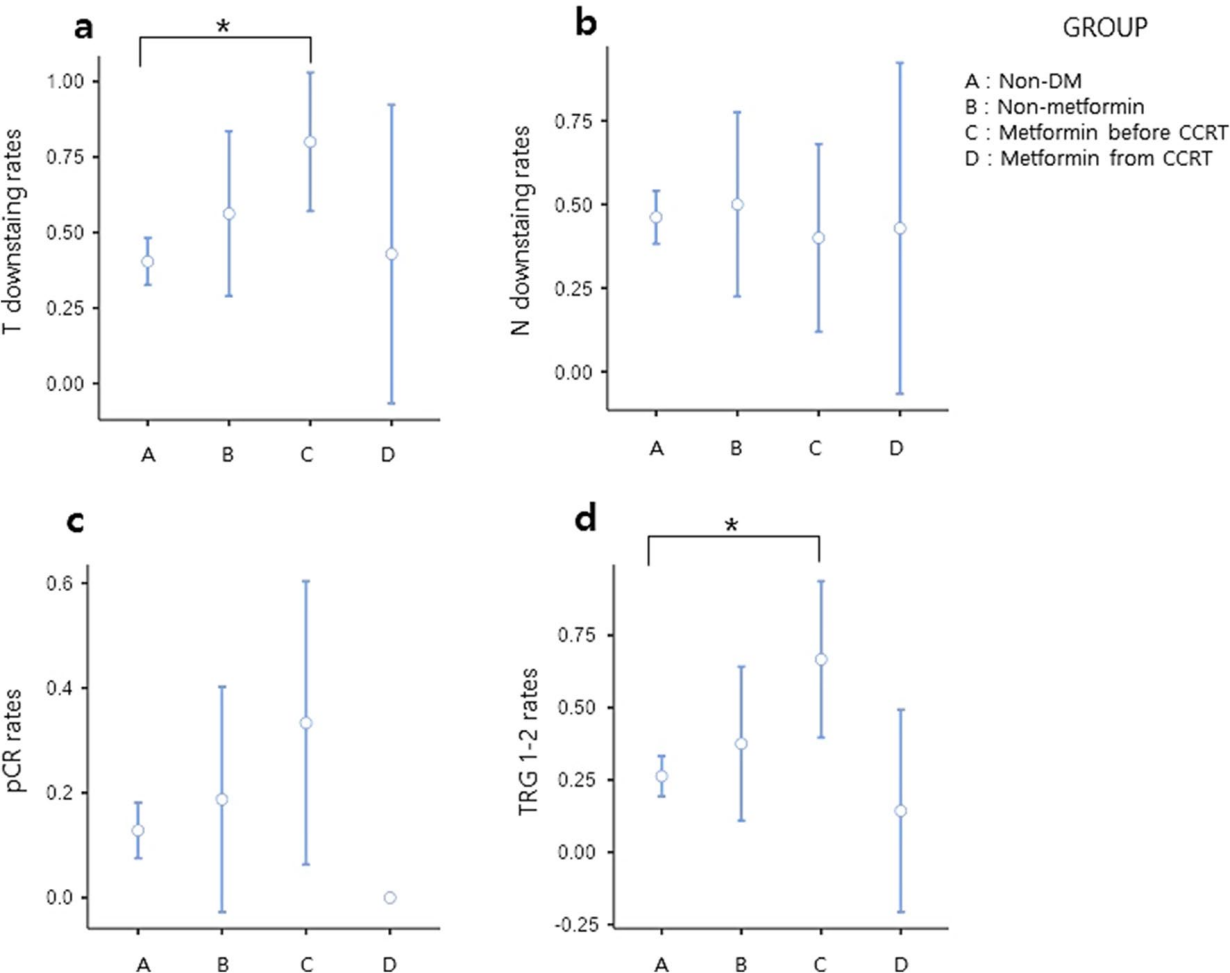
Tumor response rates between groups. (**a**) T downstaging, (**b**) N downstaging, (**c**) pathologic complete response, (**d)** tumor regression grade, * *p* < 0.005.

Tumor responses among the 4 groups: T-downstaging, N-downstaging, pTRG, and pCR were evaluated through histopathology after surgery. There were no significant differences in clinical T and N stages, and postoperative ypT and ypN stages among the four groups. There was no significant difference between the 4 groups in N-downstaging. However, the rate of T-downstaging was significantly higher in the group administered metformin before the initiation of CCRT (80.0%, *p* < 0.02). In addition, the good response rate of pTRG was also observed significantly higher in the group administered metformin before the initiation of CCRT (66.7%, *p* < 0.008). Tumor responses of the groups are described in Table 2. Especially, the rate of T-downstaging and the good response of pTRG was significantly higher in the group receiving metformin before the initiation of CCRT than in the non-diabetic group (Fig. 2).

**Table 2:** Tumor response by groups.

	Non-diabetic N = 156	Non-Metformin N = 32	Metformin before CCRT N = 30	Metformin from CCRT N = 14	*p* value
**ypStage**					0.951
pCR	18 (11.5%)	6 (18.8%)	4 (13.3%)	0 (0.0%)	
Stage ypI	34 (21.8%)	10 (31.2%)	12 (40.0%)	4 (28.6%)	
Stage ypIIA	59 (37.8%)	6 (18.8%)	6 (20.0%)	8 (57.1%)	
Stage ypIIB	1 (0.6%)	0 (0.0%)	0 (0.0%)	0 (0.0%)	
Stage ypIIIA	8 (5.1%)	0 (0.0%)	2 (6.7%)	0 (0.0%)	
Stage ypIIIB	35 (22.4%)	10 (31.2%)	4 (13.3%)	2 (14.3%)	
Stage ypIIIC	1 (0.6%)	0 (0.0%)	2 (6.7%)	0 (0.0%)	
cT*					0.047
3	153 (98.1%)	30(93.8%)	26 (86.7%)	12 (85.7%)	
4	3 (1.9%)	2 (6.2%)	4 (13.3%)	2 (14.3%)	
**ypT**^**†**^					0.405
0	20 (12.8%)	6 (18.8%)	10 (33.3%)	0 (0.0%)	
1	9 (5.8%)	4 (12.5%)	2 (6.7%)	4 (28.6%)	
2	32 (20.5%)	6 (18.8%)	10 (33.3%)	0 (0.0%)	
3	94 (60.3%)	8 (50.0%)	8 (26.7%)	10 (71.4%)	
4a	1 (0.6%)	0 (0.0%)	0 (0.0%)	0 (0.0%)	
**cN**^**‡**^					0.987
0	65 (41.7%)	16 (50.0%)	12 (40.0%)	6 (42.9%)	
1	22 (14.1%)	2 (6.2%)	4 (13.3%)	2 (14.3%)	
2	69 (44.2%)	14 (43.8%)	14 (46.7%)	6 (42.9%)	
ypN*^†^					0.757
0	112 (71.8%)	22 (68.8%)	22 (73.3%)	12 (85.7%)	
1a	15 (9.6%)	6 (18.8%)	2 (6.7%)	0 (0.0%)	
1b	12 (7.7%)	4 (12.5%)	2 (6.7%)	0 (0.0%)	
1c	3 (1.9%)	0 (0.0%)	0 (0.0%)	0 (0.0%)	
2a	13 (8.3%)	0 (0.0%)	2 (6.7%)	2 (14.3%)	
2b	1 (0.6%)	0 (0.0%)	2 (6.7%)	0 (0.0%)	
TRG*^†§^					0.008
Poor	115 (73.7%)	20 (62.5%)	10 (33.3%)	12 (85.7%)	
Good	41 (26.3%)	12 (37.5%)	20 (66.7%)	2 (14.3%)	
**pCR**					0.107
No	136 (87.2%)	26 (81.2%)	20 (66.7%)	14 (100.0%)	
Yes	20 (12.8%)	6 (18.8%)	10 (33.3%)	0 (0.0%)	
**T downstaging**					0.022
No	93 (59.6%)	14 (43.8%)	6 (20.0%)	8 (57.1%)	
Yes	63 (40.4%)	18 (56.2%)	24 (80.0%)	6 (42.9%)	
**N downstaging**					0.951
No	84 (53.8%)	16 (50.0%)	18 (60.0%)	8 (57.1%)	
Yes	72 (46.2%)	16 (50.0%)	12 (40.0%)	6 (42.9%)	

Data are n (%), TRG = tumor regression grade, pCR = pathologic complete response, cT* = Clinical Tumor stage; ypT† = Pathological tumor stage; cN‡ = Clinical Node stage; ypN*† = Pathological Node stage; TRG*†§ = Mandard grade (1–2; good, 3–5; poor).

Univariate and multivariate analyses of logistic regression models were performed to identify the factors predicting the tumor responses. There was no difference in tumor response between 5-FU and capecitabine preoperative chemotherapy (*p* = 0.2). There was no significant difference in tumor response according to total radiation dose (*p* = 0.1). Furthermore, there was no statistical difference in T-downstaging (*p* = 0.2), N-downstaging (*p* = 0.8), pCR (*p* = 0.3), and pTRG (*p* = 0.2) according to the dose of metformin. In univariate logistic regression analysis; HbA1c, vascular invasion (VI), ypT, and cN were significant factors for predicting pCR; cT, cN, and ypT were independent factors related to N-downstaging; Metformin administration before the initiation of CCRT, low histological grade, VI, lymphatic invasion (LI), cT, cN, and ypN were independent factors associated with T-downstaging; Metformin administration before the initiation of CCRT, VI, LI, cN, ypT, T-downstaging, and N-downstaging were significant factors for predicting good pTRG. Multivariate analysis was performed with predictors related to the tumor response in the univariate analysis (Table 3). Metformin administration before the initiation of CCRT was a significant factor in predicting T-downstaging (OR 10.31, 95% CI 1.76–102.08, *p* = 0.02) and good response of pTRG (OR 12.55, 95% CI 2.38–80.24, *p* = 0.004).

**Table 3:** Multivariable logistic regression analysis for tumor response.

Variables	OR	95% CI		*p* value
**T downstaging**				
Neoadjuvant metformin	10.31	1.76	102.08	0.02
Preoperative CEA	0.96	0.89	1.02	0.23
Histologic grade	15.78	2.52	34.167	0.01
VI	0.48	0.1	2.02	0.32
LI	0.44	0.08	2.3	0.33
cT	24.53	1.43	17.875	0.08
cN	0.88	0.61	1.29	0.51
**N downstaging**				
Age	1.03	0.98	1.07	0.27
cT	1.84	0.19	4.977	0.65
ypT	0.73	0.31	1.69	0.45
cN	17.71	9.22	39.36	< 0.001
pCR	0.66	0.06	7.86	0.73
**pCR**				
Age	0.96	0.71	1.17	0.72
HbA1c	20.22	2.29	3.26	0.07
Preoperative CEA	0.64	0.23	1.32	0.26
Location of tumor	1.74	0.3	30.65	0.60
VI	0.3	0.01	8.353	0.77
ypT	0.1	0.002	0.02	0.01
cN	0.2	0.01	1.31	0.15
**TRG**				
Age	0.99	0.95	1.03	0.61
Neoadjuvant metformin	12.55	2.38	80.24	0.004
Preoperative CEA	1.02	0.92	1.1	0.70
VI	0.35	0.03	2.75	0.36
LI	0.41	0.02	4.26	0.50
ypT	0.09	0.03	0.21	< 0.001
cN	1.27	0.35	4.74	0.71

VI = vascular invasion, LI = lymphatic invasion, CEA = carcinoembrionic antigen, TRG = tumor regression grade, CI = confidence interval.

### Survival analysis

**Figure 3:**
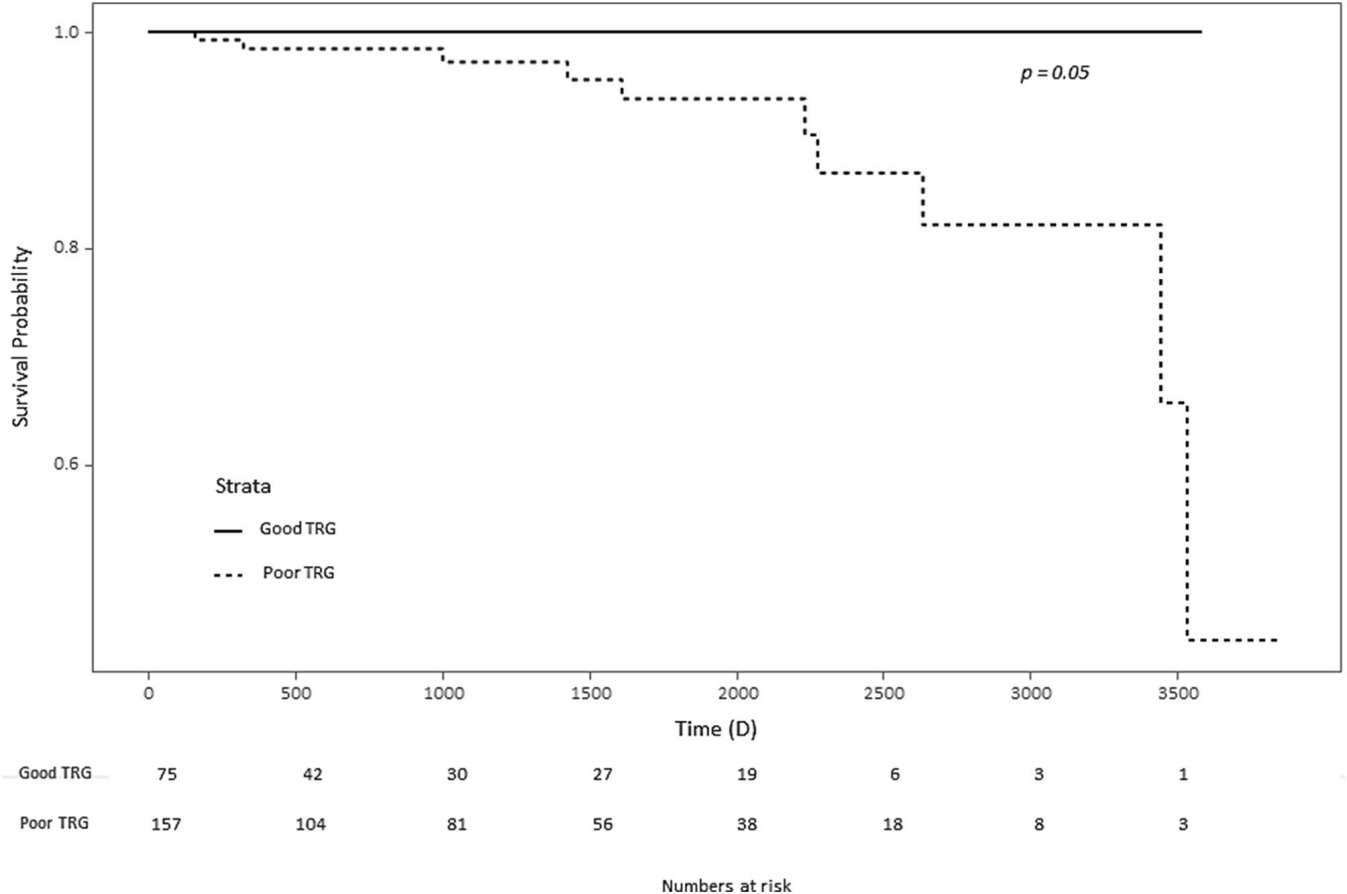
The Kaplan–Meier curve for overall survival according to tumor regression grade.

The follow-up period for survival was at least 4 years. In the survival analysis, there was no significant difference in the overall survival between the 4 groups. There was no difference in the overall survival rate for the timing of metformin administration for CCRT (*p* = 0.4). There was no significant difference in the overall survival rate according to CRM status (*p* = 0.8) and adjuvant chemotherapy regimen (*p* = 0.2). There was a trend of increased survival rate in the good pTRG group, but there was no statistically significant difference in the survival rate between good and poor pTRG (Fig. 3). Because the total number of events was insufficient, the cox proportional hazards model for survival factors analysis could not be performed.

## Discussion

Preoperative CCRT for rectal cancer is an important treatment for inducing good tumor response and ultimately achieving pCR for reducing recurrence rate. Furthermore, pathological complete response (pCR) after CCRT achieves a lower rate of local recurrence (LR)^14^ and correlates with better survival after surgery^3^. However, Response to preoperative CCRT is variable and only 10–20% of patients have a pathological complete response without residual tumor, and the factors that influence tumor response are not well understood^4^. Several randomized control trials (RCTs) have demonstrated that the local recurrence rate decreases in patients receiving preoperative CCRT for rectal cancer^15–17^. If a patient fails to achieve an adequate tumor response, the patient's prognosis may be poor. Therefore, studies to find alternatives or adjuvants that can induce better tumor response are attracting attention.

Some studies have reported the effectiveness of metformin as an anticancer therapy and anticancer adjuvant for various cancers^5,6^. Several recent studies show that metformin improves tumor response after preoperative CCRT for rectal cancer^9,10^. Retrospective studies have suggested that metformin use is associated with N-downstaging, pCR, and good TRG^9,10,18^. Several researchers have conducted studies on metformin, but there are no clinical studies on the timing and dose of metformin for tumor response of preoperative CCRT. This study showed that the timing of metformin administration for CCRT may be related to the tumor response. Indeed, a study has shown that the formation of aberrant crypt foci (ACF) in the rectum is inhibited in patients receiving metformin for 1 month^19^. Additionally, Zhao et al. reported that when metformin was administered at 1500 mg/day, the number of ACFs significantly reduced in the patient group taking metformin for 6 months compared to when metformin was administered for 3 months^20^. In our study, patients who took metformin before the initiation of CCRT showed better tumor response than those who took metformin from the initiation of CCRT. In several studies, the rate of stage reduction due to metformin was 45–70%^9,10^. In our study, the rates of T-downstaging, N-downstaging, and good pTRG in the underlying metformin usage group were higher than in the other groups (80%, 40%, 66.7%, respectively). In particular, the rates of T-downstaging and good pTRG increased significantly in the underlying metformin usage group (*p* = 0.02, *p* = 0.008, respectively). In other studies, the pCR rate was 20–25%^9,10^, but in this study, the pCR rate of 33.3% was observed in patients who took metformin before the initiation of CCRT. In a multivariate analysis to identify predictors of tumor responses, metformin administration before the initiation of CCRT was found to be a significant predictor for predicting T-downstaging and good pTRG. These results suggest that neoadjuvant metformin administration may have an intensive effect in addition to CCRT.

The importance of the Akt phosphorylation and consequent activation in conferring resistance to radiation therapy has been shown, which explains the molecular mechanisms that determine poor response to radiation therapy^21^. Metformin interferes with the mitochondrial respiratory complex 1 and decreases the effectiveness of intracellular ATP. Reduction of ATP indirectly increases the activity of AMPK, an inhibitor of the PI3K/Akt/mTOR pathway, resulting in an overall decrease in the mTOR pathway^22,23^. In the cell line treated with metformin for more than 24 h, AMPK was significantly increased, but within 2 h, AMPK was not increased^24^. In addition, phosphorylation of Akt did not decrease at 2 h after metformin treatment, but it was removed after 24 h^24^. Treatment with metformin inhibits the PI3K/Akt/mTOR pathway, inhibiting protein synthesis and cell growth, resulting in anticancer activity. The above results suggest that the timing of metformin administration may affect the treatment response, as shown in our study.

One study shows that metformin increases survival rates in patients with colorectal cancer^9^. However, in the ECR-PHARMO cohort, it was reported that the benefit of overall survival in colorectal cancer was not associated with metformin^25^. In our study, the dose and timing of metformin did not show a significant improvement in overall survival. Although this study showed a trend of increased survival rate in the good pTRG group, it is likely that the patient's overall sample size and death events were not enough to derive significant results.

There are several limitations to this study. First, there are limitations of retrospective studies and small sample sizes. Second, since preoperative staging was based on imaging studies, it is inevitable to limit the accuracy of tumor response. However, to our knowledge, this study is the first clinical study on the neoadjuvant effect of metformin for tumor response after preoperative CCRT. A synergistic effect between the neoadjuvant metformin and CCRT can be expected based on this study. In addition, the potential effects of neoadjuvant metformin may affect the choice of a short course or long course radiation therapy. In the future, long-term, large-scale, well-designed randomized controlled trials are needed in the setting of patients with the adjustment for detailed relevant clinical and molecular factors. Furthermore, large-scale studies of dose, timing, and possible effects of metformin on radiation therapy in the general population without DM will be helpful for the treatment of rectal cancer.

In conclusion, **i**n patients with rectal cancer who underwent preoperative CCRT and curative surgery, underlying metformin usage was significantly associated with good tumor response. This study suggests that neoadjuvant metformin can improve tumor response to radiation therapy in patients with rectal cancer. This study is of value in developing therapeutic strategies to improve tumor response in patients with rectal cancer.

## Data Availability

Data are available from the authors upon reasonable request and with permission.
